# The sputum microbiome associated with different sub-types of AECOPD in a Chinese cohort

**DOI:** 10.1186/s12879-020-05313-y

**Published:** 2020-08-18

**Authors:** Juan Wang, Jianmin Chai, Lina Sun, Jiangchao Zhao, Chun Chang

**Affiliations:** 1 Department of Respiratory and Critical Care Medicine, Peking University Third Hospital, Beijing, China; 2 Department of Animal Science, Division of Agriculture, University of Arkansas, Fayetteville, AR 72701 USA

**Keywords:** Sputum microbiome, AECOPD, Eosinophilic AECOPD, Subtypes, Biomarkers

## Abstract

**Background:**

Chronic obstructive pulmonary disease (COPD) is one of the most prevalent diseases worldwide. Episodes of acute exacerbations of COPD (AECOPD) are associated with disease severity and progression. Although substantial progress has been made in understanding the dynamics of AECOPD, little is known about the sputum microbiome of AECOPD in the Chinese population.

**Methods:**

In this study, we characterized the sputum microbiomes from sputum specimens collected from healthy controls (*n* = 10), stable (*n* = 4), AECOPD (*n* = 36), and recovery (*n* = 18) stages by sequencing the V3-V4 region of the 16S rRNA gene with a HiSeq sequencer.

**Results:**

*Streptococcus* was the most dominant genus among all the different types of sputum. A random forest model was developed to identify bacterial taxa that differentiate AECOPD samples from others. Most of the top predictors, except *Pseudomonas*, were less abundant in AECOPD samples. We also developed random forest models to differentiate subtypes of AECOPD based on blood eosinophil counts, the frequency of AECOPD, and sputum eosinophils. Bacterial taxa associated with *Pasteurellaceae*, *Fusobacterium*, *Solobacterium*, *Haemophilus*, *Atopobium*, *Corynebacterium* and *Streptococcus*, were enriched in the sputum microbiomes of eosinophilic AECOPD. Random forest models also demonstrate that a total of 2 bacterial OTUs were needed to differentiate frequent from non-frequent AECOPDs, and 23 OTUs were enough to accurately predict sputum-eosinophilic (sputum eosinophilic concentration ≥ 3%) AECOPD.

**Conclusion:**

This study expanded our understanding of the sputum microbiome associated with different subtypes and clinical status of patients with AECOPD in a Chinese cohort, which provides insights into novel and more targeted management of the different subtypes of AECOPD.

## Background

Chronic obstructive pulmonary disease (COPD), one of the most prevalent respiratory diseases, is currently the fourth leading cause of mortality worldwide, according to the World Health Organization (WHO) consensus reports, and is forecasted to rank as the third cause of mortality by 2020 [1]. Episodes of acute exacerbations of COPD (AECOPD) and the sudden worsening of symptoms, represent substantial social and medical burdens, and are major causes of COPD-related morbidity and mortality [2, 3]. One major etiological factor of AECOPD is bacterial colonization [4, 5]. Over 50% of AECOPD cases are caused by a bacterial infection. Changes in the lung microbiota associated with enhanced airway inflammation and disease progression have been demonstrated [6–9]. The overgrowth of pathogens including *Pseudomonas aeruginosa*, *Haemophilus influenzae*, *Streptococcus pneumoniae*, and *Moraxella catarrhalis* in the human respiratory tract and the subsequent onset of AECOPD were also reported [10]. In spite of this substantial progress, relatively little has been reported about the sputum microbiome of AECOPD in the Chinese population [10–12].

Recently, blood eosinophil counts have been used as a biomarker of AECOPD. Patients with blood eosinophil count ≥2% responded better to systemic corticosteroid treatment [13, 14] and were significantly less likely to have exacerbations when inhaled corticosteroids were applied [15]. Yun and colleagues showed that patients with moderate-to-severe COPD and ≥ 300 blood eosinophils/μl had a greater risk of AECOPDs [16]. It is estimated that 20–40% of patients with stable COPD have eosinophilic airway inflammation. This airway eosinophilia increased upon AECOPD [17, 18]. Despite the importance of eosinophilic AECOPD, little is known about the microbiome of this subtype of AECOPD [8].

Although it is prone to oral microbiome contamination given the topological continuity of the oral cavity and the lower respiratory tract, sputum has been widely used as a surrogate to study the lung microbiome in different respiratory diseases [19, 20]. The objectives of this study were to characterize the AECOPD sputum microbiome in a Chinese population and to determine whether specific sputum microbiome biomarkers could be identified in order to differentiate eosinophilic from other subtypes of AECOPD. To this end, we examined 68 sputum samples collected from healthy subjects, COPD patients in the stability, exacerbation, and recovery periods with different eosinophil counts. We found that *Streptococcus* was the most dominant genus among all the different types of sputum in this Chinese cohort, and we also developed random forest models that differentiate the sputum microbiome of eosinophilic AECOPD from those of other subtypes.

## Methods

This study was carried out in accordance with the recommendations of the Ethics Committee of Peking University Third Hospital with written informed consent from all subjects. All subjects gave written informed consent in accordance with the Declaration of Helsinki. The protocol was approved by the Ethics Committee of Peking University Third Hospital (#196–03).

### Subject enrollment and metadata

From May 2016 to March 2018, patients presenting AECOPD were admitted to the Respiratory Department in Peking University Third Hospital as well as ten healthy controls from outpatient clinics were enrolled. For patients admitted more than once during the study period, only the first admission was included. Inclusion criteria were: COPD diagnosis or acute exacerbation that met the GOLD definition [1]. Patients with a history of other respiratory illnesses, such as acute asthma, pulmonary tuberculosis, sleep apnea syndrome, bronchiectasis, lung cancer, or interstitial lung disease, were excluded. All participants gave written informed consent.

Clinical data, including demographic data, tobacco exposure, indices of lung function, exacerbation frequency in the previous year, and admission symptoms were collected (Table 1). On the day of admission, the Anthonisen type of AECOPD was determined based on symptoms before starting treatment [21]. The frequency of exacerbation was determined by the numbers of hospitalizations caused by AECOPD in the previous year.

**Table 1:** Cohort characteristics

Characteristics	COPD (*n* = 47)	Control (*n* = 10)
Male gender (%)	40(85.1%)	3(30%)
Age (years)	72 ± 9.8	57 ± 10.7
BMI (kg/m2)	22.9 ± 4.9	21.2 ± 3.3
Smoker	44	4
FEV1 (% pred)	46.7 ± 18.5	100 ± 14.3
Eosinophils percentage (Eos%) in blood (n)	51	
≥ 2	19	
<2	32	
Eosinophils% in sputum (n)	55	10
≥ 3	10	1
<3	45	9
Frequency of exacerbation in previous year (n)	40	
≥ 2	7	
<2	33	

Health Control: subjects without any clinical signs; AECOPD: acute exacerbations of chronic obstructive pulmonary disease; BMI: Body mass index; FEV: Forced expiratory volume

In order to examine the difference in the sputum microbiome between different AECOPD phenotypes, participants were divided into subgroups according to with or without hospital admissions due to exacerbation in the previous year, eosinophils percentage (Eos%) in blood ≥2% or <2%, Eos% in sputum ≥3% or <3%. Among the 36 AECOPD samples, five were collected from patients without antibiotic treatment, two were from patients without known medical records about antibiotic treatment, and the remaining were collected after patients were treated with antibiotics. Since most of our patients were moderate to severe, they almost always received therapeutic doses of antibiotics when they had symptoms of AECOPD and were hospitalized when symptoms became worse. Since antibiotic-treated AECOPD microbiomes were no different from those without antibiotics (Figure S1), we did not distinguish antibiotic-treated vs. non-treated AECOPD samples in subsequent analysis.

### Sample collection and storage

Each subject was asked to rinse his/her mouth and posterior oropharynx by swishing and gargling with a 3% hypertonic saline solution before sampling. Either spontaneous or induced sputa were collected from patients. For induced sputum collection, patients were nebulized with a 3% saline solution to expectorate enough sputum within 30 min. Sputum specimens were homogenized by incubation with 0.4% dithiothreitol (DTT, Millipore, Canada) with shaking at 37 °C for 30 min. An aliquot of the sputum solution was then used for sputum cytology, and the remainder was divided into 0.5 ml aliquots and stored at − 80 °C for DNA extraction.

A total of 68 specimens were assigned to one of four clinical states based on medical record review: 1. healthy control (*n* = 10), 2. stable (stable, *n* = 4, over eight weeks free of an AECOPD), 3. exacerbation (AECOPD, *n* = 36, defined according to Anthonisen criteria [21], and/or healthcare utilization [22]), 4. recovering from exacerbation treatment (recovery, *n* = 18, two weeks after treatment of AECOPD).

### DNA extraction and sputum microbiome sequencing

Homogenized sputum samples from frozen stocks were thawed on ice, and microbial DNA was extracted with QIAamp® DNA Microbiome kit (QIAGEN, Germany) according to the manufacturers’ instructions. The V3-V4 region of the bacterial 16S rDNA was amplified with indexes and adaptors-linked universal primers (341F: ACTCCTACGGGAGGCAGCAG, 806R: GGACTACHVGGGTWTCTAAT). PCR was performed using the KAPA HiFi Hotstart PCR kit high fidelity enzyme. Amplicon libraries were quantified by a Qubit 2.0 Fluorometer (Thermo Fisher Scientific, Waltham, US) and then sequenced on the Illumina HiSeq platform (Illumina, San Diego, US) for paired-end reads of 250 bp at the Realbio Genomics Institute (Shanghai, China). To exclude contamination from the reagents and environment, we included two negative controls (water), with one in the DNA extraction step and the other in PCR amplification. No detectable bands were observed in agarose gels from the negative controls.

### Sequence and data analysis

We used the mothur software package (v.1.39.1) [23] to analyze the 16S rRNA HiSeq data. After removing ambiguous bases, the sequences were aligned with the SILVA reference database (Full length sequences and taxonomy references release 132, http://www.arb-silva.de/). Then, sequences were further de-noised, and chimeras were detected and removed by the UCHIME algorithm [24]. The high quality sequences were clustered into operational taxonomic units (OTUs) at the 97% similarity level and were classified against the Ribosomal Database Project [25]. Sequences were rarefied to the smallest number of reads (22450) to minimize the influence of sequencing depth on the measures of alpha and beta diversity. Shannon Index and the number of observed OTUs were calculated to estimate alpha diversity. The Bray-Curtis and Jaccard distance metrics were calculated to explore the dissimilarities in community structure and membership, respectively. The ANalysis Of SIMilarity (ANOSIM) test was used to examine the statistical significance of differences in beta diversity.

Random forest was performed to identify the top microbiome signatures to differentiate groups or AE subtypes. R package ‘AUCRF’ (v.1.1) was used to perform random forest process and select variables based on optimizing the area-under-the Receiver Operator Characteristic (ROC) curve (AUC) of the Random Forest [26]. The alpha diversity (Shannon Index, Chao1 and the number of observed OTUs) and the relative abundance of all the OTUs (*n* = 1395) were included in the model for feature selection. The ‘importance’ and ‘proximity’ parameters were set ‘True’, and ‘ntree’ was set at 10,000 in the model. A 10-fold cross validation analysis was performed and repeated 20 times to calculate the probability of each selected variable. The number of optimal variables with the greatest sensitivity and specificity was calculated using the ‘pROC’ package (v.1.13). Thus, a variable importance plot was produced according to the importance scores (Mean Decrease in Accuracy, MDA) of selected features and their boxplots of selected features were drawn in R. Raw data were deposited into the SRA database with accession number PRJNA647843.

## Results

### The overall sputum microbiome of AECOPD

**Fig. 1:**
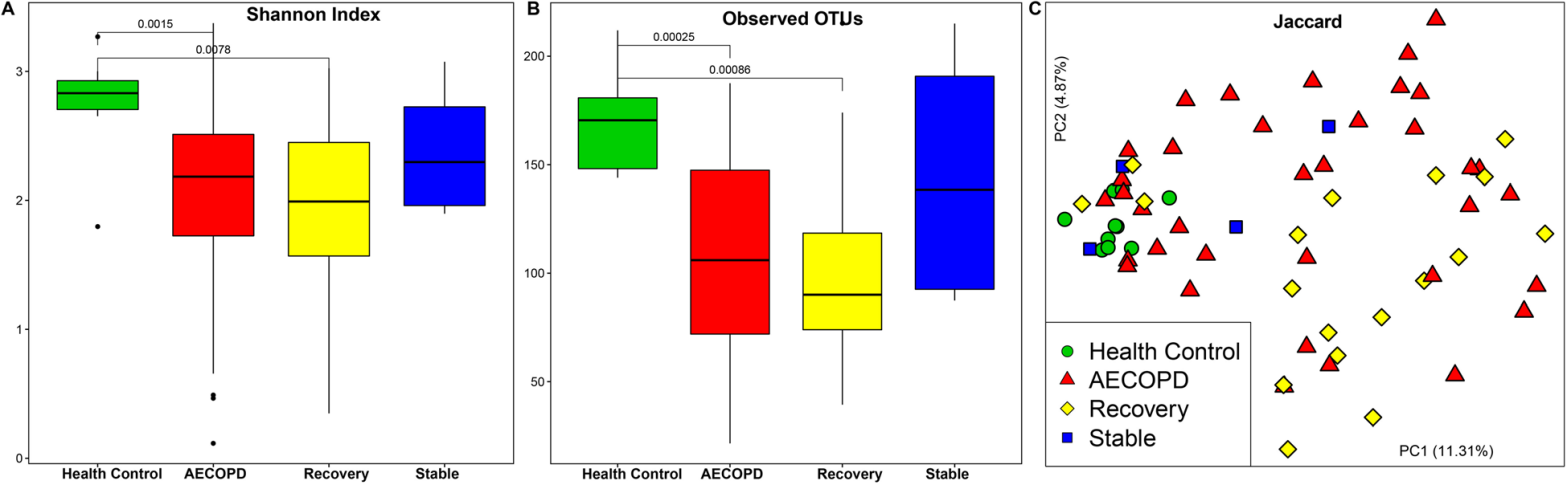
Alpha and beta diversities of the sputum microbiome in healthy controls (*n* = 10), AECOPD (*n* = 36), recovery (*n* = 18) and stable samples (*n* = 4): Community diversity (Shannon Index) and richness (number of observed OTUs) (**a** and **b**). The dark horizontal lines within the bar of A and B were the median values of each group. After the Kruskal–Wallis test is significant, a post-hoc analysis (Wilcoxon signed-rank test) was performed to determine which group of the independent variable differ from each other group. A and B indicated that either AECOPD or Recovery subjects is significantly less diverse and richness than the healthy control. Principal Coordinate Analysis of the sputum microbiome membership based on the Jaccard distance matrix (**c**). Each point represents 1 subject with Health Control as green circle, AECOPD as red triangle and Stable as blue square. Regarding beta diversity measures, significant differences in community membership between AECOPD vs healthy controls (ANOSIM, R = 0.43, *P* < 0.05), AECOPD vs stable (ANOSIM, R = 0.29, *P* < 0.05), healthy control vs recovery (R = 0.47, *P* < 0.05) and healthy control vs stable (R = 0.38, *P* < 0.05) were detected. Health Control: subjects without any clinical signs; AECOPD: acute exacerbations of chronic obstructive pulmonary disease; Recovery: the patient recovering from exacerbation treatment; Stable: stable period over 8 weeks free of an AECOPD

We first analyzed all the sputum microbiomes (*n* = 68) collected. A significant decrease in sputum microbial diversity was observed in the samples collected from AECOPD patients compared to those collected from healthy controls (*n* = 10). Although not statistically significant, AECOPD samples had lower diversity than stable samples, likely due to accumulated exposure to antibiotics and corticosteroids. Samples collected from the recovery stage had the smallest alpha diversity measures (Fig. 1a and b). Regarding beta diversity measures, significant differences in community membership between AECOPD vs. healthy controls (ANOSIM, R = 0.43, *P* < 0.05), AECOPD vs. stable (ANOSIM, R = 0.29, *P* < 0.05), healthy control vs. recovery (R = 0.47, *P* < 0.05) and healthy control vs. stable (R = 0.38, *P* < 0.05) were detected. No significant difference in community membership between AECOPD vs. recovery (R = -0.05, *P* = 0.78), and stable vs. recovery (R = 0.17, *P* = 0.15) was observed (Fig. 1c). With respect to community structure, significant differences between AECOPD vs. stable (R = 0.22, *P* < 0.05), healthy control vs. recovery (R = 0.44, *P* < 0.05), and stable vs. recovery (R = 0.43, *P* < 0.05) were revealed by the PCoA plot based on Bray-Curtis distance (Figure S2).

### Sputum microbiome signatures of AECOPD

**Fig. 2:**
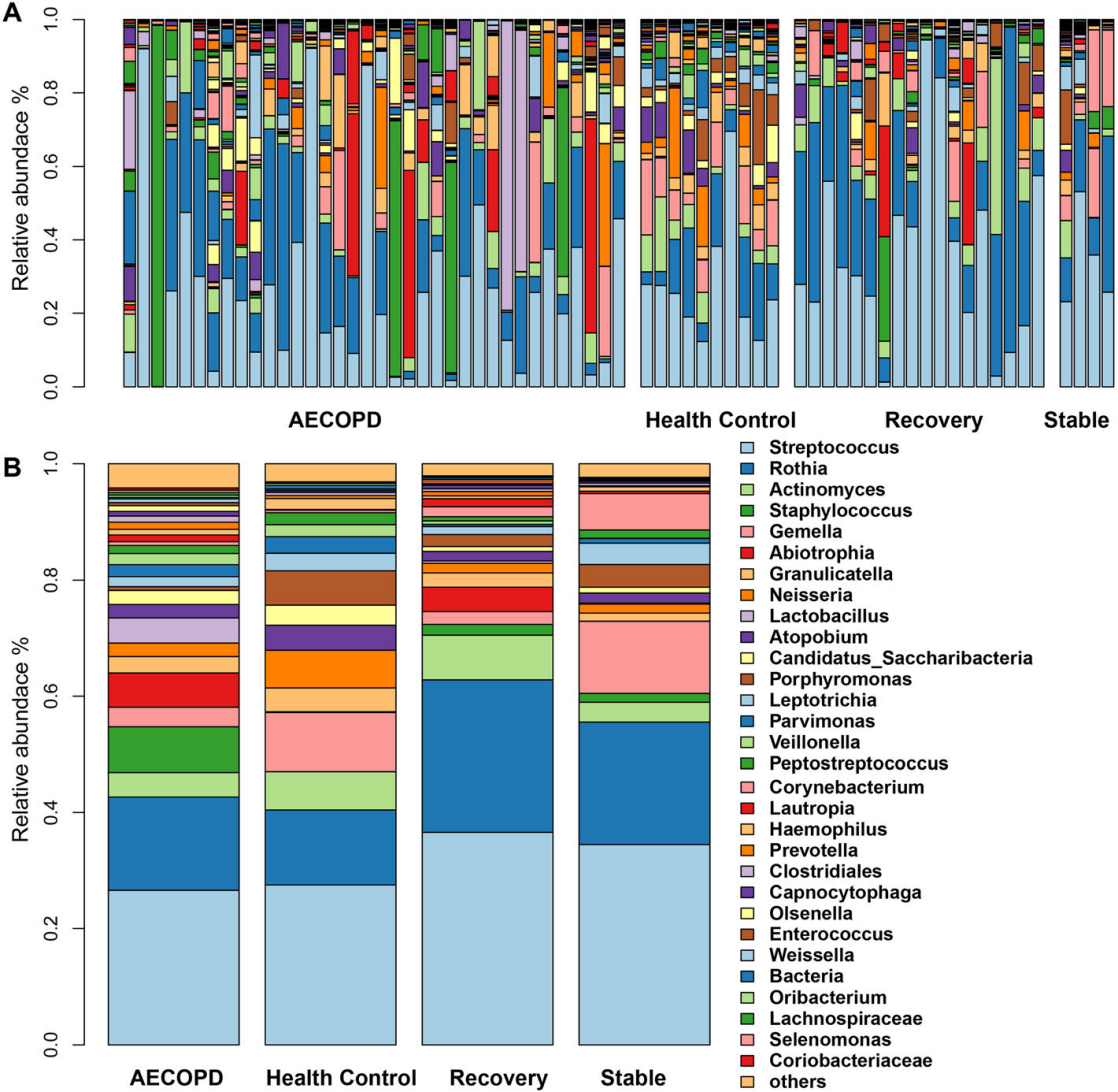
The sputum microbiome at the genus level. Each bar shows the relative abundance of individual (**a**) or average (**b**) samples collected at AECOPD, healthy controls, recovery and Stable. Across all samples, the sum of average relative abundance of top30 genera was over 96%. Health Control: subjects without any clinical signs; AECOPD: acute exacerbations of chronic obstructive pulmonary disease; Recovery: the patient recovering from exacerbation treatment; Stable: stable period over 8 weeks free of an AECOPD

Next, we examined the sputum microbiome composition of AECOPD. At the phylum level, the AECOPD sputum microbiome was dominated by Firmicutes (60.99%), followed by Actinobacteria (25.75%) and Proteobacteria (5.59%). Similar patterns were observed in the sputum microbiome collected at stable (Firmicutes 53.95%, Actinobacteria 33.47%, Bacteroidetes 4.69%, Proteobacteria 2.91%) and recovery (Firmicutes 51.46%, Actinobacteria 37.81%, Proteobacteria 4.16%) stages of the COPD patients as well as in healthy controls (Firmicutes 52.06%, Actinobacteria 24.80%, Proteobacteria 8.66%, Bacteroidetes 7.01%) (Figure S3). At the genus level, the top five genera of the AECOPD sputum microbiome were *Streptococcus* (26.59%), *Rothia* (16.07%), *Staphylococcus* (7.83%) *Abiotrophia* (5.89%), and *Lactobacillus* (4.34%), which accounted for over 60.27% of the sequences. *Streptococcus* (27.52%), *Rothia* (12.93%), *Lactobacillus* (10.13%), *Staphylococcus* (6.60%), and *Granulicatella* (6.49%) were the top five genera in healthy controls. Similarly, samples collected at the recovery stage were dominated by *Streptococcus* (36.60%), *Rothia* (26.20%), *Staphylococcus* (7.71%), *Actinomyces* (4.20%), and *Gemella* (2.46%), whereas in the stable samples, *Streptococcus* (34.49%), *Rothia* (21.04%), *Lactobacillus* (12.43%), *Lautropia* (6.26%), and *Parvimonas* (3.91%) were the top five genera (Fig. 2, Table S1).

**Fig. 3:**
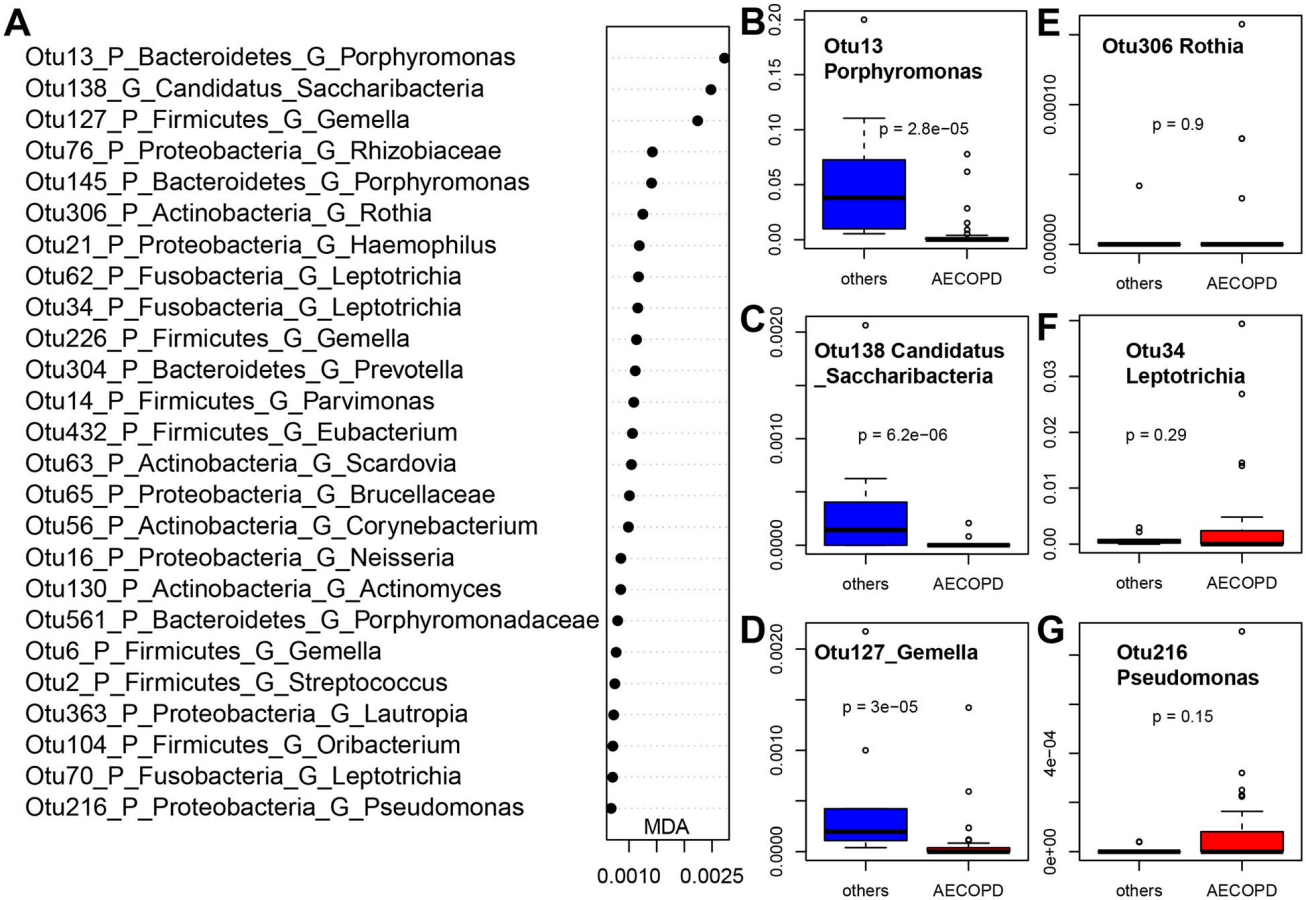
Bacterial OTUs that differentiate AECOPD (*n* = 36) vs other samples (*n* = 32): Mean Decrease in Accuracy (MDA) plot of the top 25 bacterial OTUs (**a**); Box plots of representative OTUs (**b**-**g**). The sputum microbiome signatures that differentiate AECOPD samples from other samples (a combination of Health Control, Recovery and Stable) were reported in this figure A: top 25 bacterial OTUs were listed based on Mean Decrease in Accuracy (MDA) from AUCRF; **b**-**g**: Box plots of relative abundance of representative OTUs, and the *p* value were calculated by using Wilcoxon test. AECOPD: acute exacerbations of chronic obstructive pulmonary disease; others: a combination of health control, recovery and stable. Health Control: subjects without any clinical signs; Recovery: the patient recovering from exacerbation treatment; Stable: stable period over 8 weeks free of an AECOPD

Random forest model revealed sputum microbiome signatures that differentiated AECOPD samples from other samples with an AUC (area under the curve) of 0.78 (specificity 0.806, sensitivity 0.688) (Figure S4). The top 25 bacterial features based on their importance in the classification of AECOPD samples are listed (Fig. 3a). Among the top 25 features, most of them were enriched in the non-AECOPD samples including common human upper respiratory tract bacteria such as *Gemella* (OTU6, 127, 226, 234), *Porphyromonas* (OTU69, 13, 145), *Haemophilus* (OTU21), *Neisseria* (OTU16, 15), *Streptococcus* (OTU30), *Candidatus_Saccharibacteria* (OTU85, 23, 169, 24, 138), and *Fusobacterium* (OTU54) (Fig. 3b-e). Among the 92 OTUs with an MDA value of 3 or greater, OTU216 (*Pseudomonas*) was more abundant in AECOPD than in other samples (Fig. 3 and Figure S5).

### The sputum microbiome of eosinophilic AECOPD

**Fig. 4:**
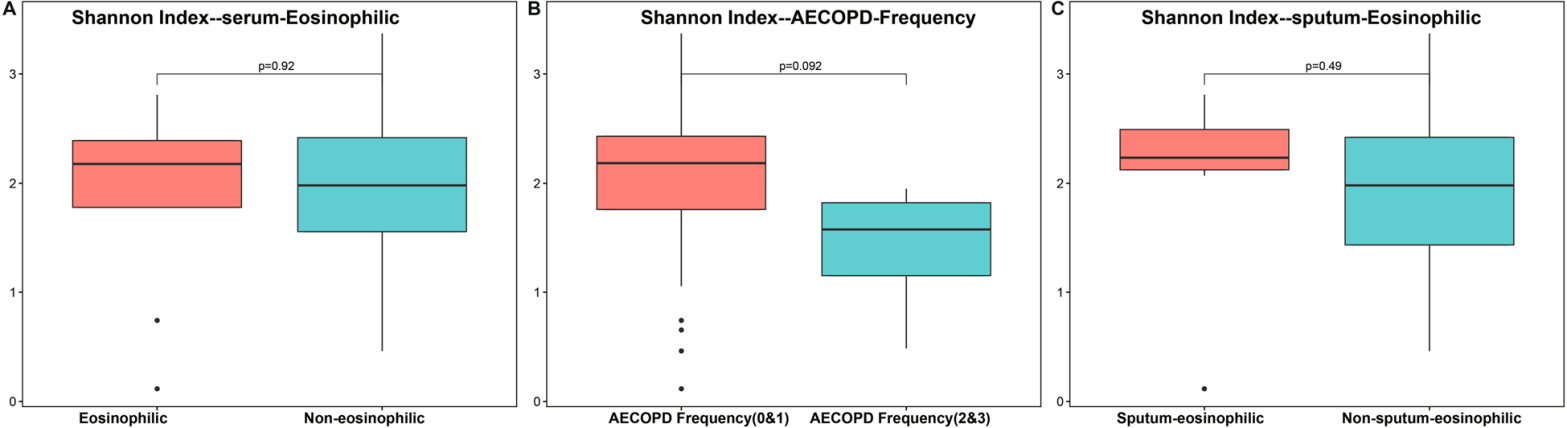
Alpha diversity of the sputum microbiomes of different subtypes of AECOPD: eosinophilic vs non-eosinophilic (**a**), frequency of AECOPD (**b**), and sputum-eosinophilic vs non-eosinophilic (**c**). In AECOPD patients, we defined the subtypes based on clinical parameters. A: eosinophils subtype: blood eosinophils < 2% as non-eosinophilic (24 samples), blood eosinophils ≥2% as eosinophilic (9 samples). **b**: AECOPD frequency (0&1) (29 samples) and AECOPD frequency (2&3) (4 samples) based on the times having AECOPD in the past 1 year. **c**: sputum eosinophils < 3% as non-sputum-eosinophilic (26 samples), sputum eosinophils ≥3% sputum-eosinophilic (7 samples). AECOPD: acute exacerbations of chronic obstructive pulmonary disease. Both serum eosinophilic and sputum eosinophilic had no significant Shannon Index. The sputum microbiome of patients with 0 or 1 AECOPD had greater alpha diversity than those with 2 or 3 AECOPDs. The *p* values were calculated by using Wilcoxon test. AECOPD: acute exacerbations of chronic obstructive pulmonary disease

**Fig. 5:**
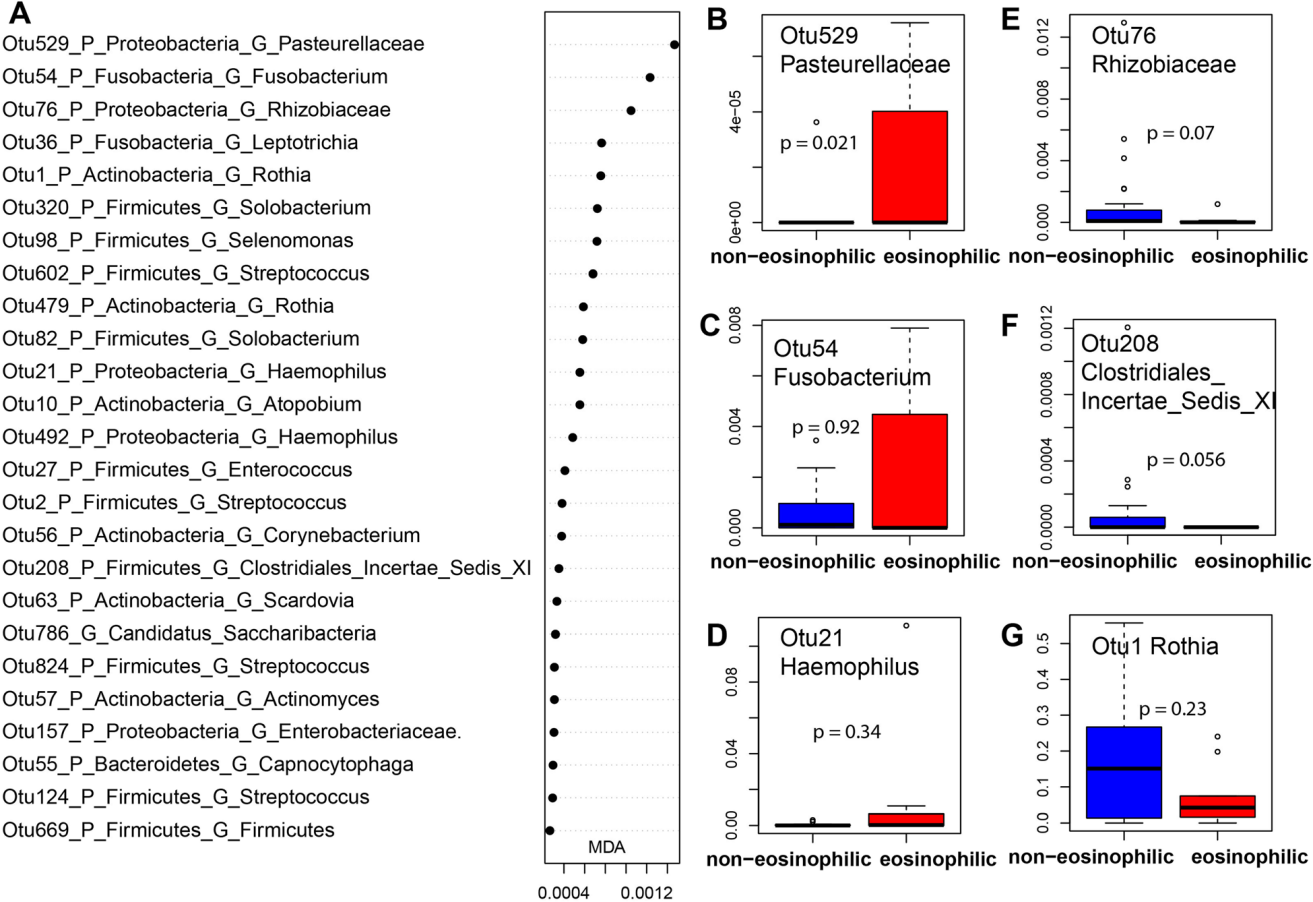
Bacterial OTUs that differentiate eosinophilic vs other AECOPDs: Mean Decrease in Accuracy plot of the top 15 bacterial OTUs (**a**); Box plots of representative OTUs (**b**-**g**). AUCRF was performed to find bacteria that differentiate serum eosinophilic and non-eosinophilic in AECOPD patients. A: top 25 bacterial OTUs were listed based on Mean Decrease in Accuracy (MDA) from AUCRF; **b**-**g**: Box plots of relative abundance of representative OTUs, and the p values were calculated by using Wilcoxon test. AECOPD: acute exacerbations of chronic obstructive pulmonary disease eosinophils subtype: blood eosinophils < 2% as non-eosinophilic (24 samples), blood eosinophils ≥2% as eosinophilic (9 samples)

Given the increasing interest in eosinophilic AECOPD, we characterized the sputum microbiome of eosinophilic AECOPD (i.e., patients with blood eosinophils ≥2%). Although no significant differences in the sputum microbiome alpha (Fig. 4a) or beta diversity (Figure S6A and B) were detected between eosinophilic AECOPD vs. non-eosinophilic AECOPD, a random forest model was able to differentiate eosinophilic from non-eosinophilic AECOPD accurately with an AUC of 0.870 (specificity 0.778, sensitivity 0.917) (Figure S4B). A total of 15 OTUs were needed to reach this accuracy. Among these OTUs, *Pasteurellaceae* (OTU529), *Fusobacterium* (OTU54), *Solobacterium* (OTU320, 82), *Leptotrichia* (OTU36,31), *Haemophilus* (OTU21), *Atopobium* (OTU10), *Corynebacterium* (OTU56), and *Streptococcus* (OTU2, OTU124) were enriched in the sputum microbiome of eosinophilic AECOPD, while OTUs such as *Rhizobiaceae* (OTU76), *Clostridiales_Incertae_Sedis*_XI (OTU208), *Enterobacteriaceae* (OTU157), *Rothia* (OTU1), *Capnocytophaga* (OTU55), and *Scardovia* (OTU63) were more abundant in patients with fewer eosinophils (< 2%) (Fig. 5 and Figure S7).

### The sputum microbiome of other subtypes of AECOPD

**Fig. 6:**
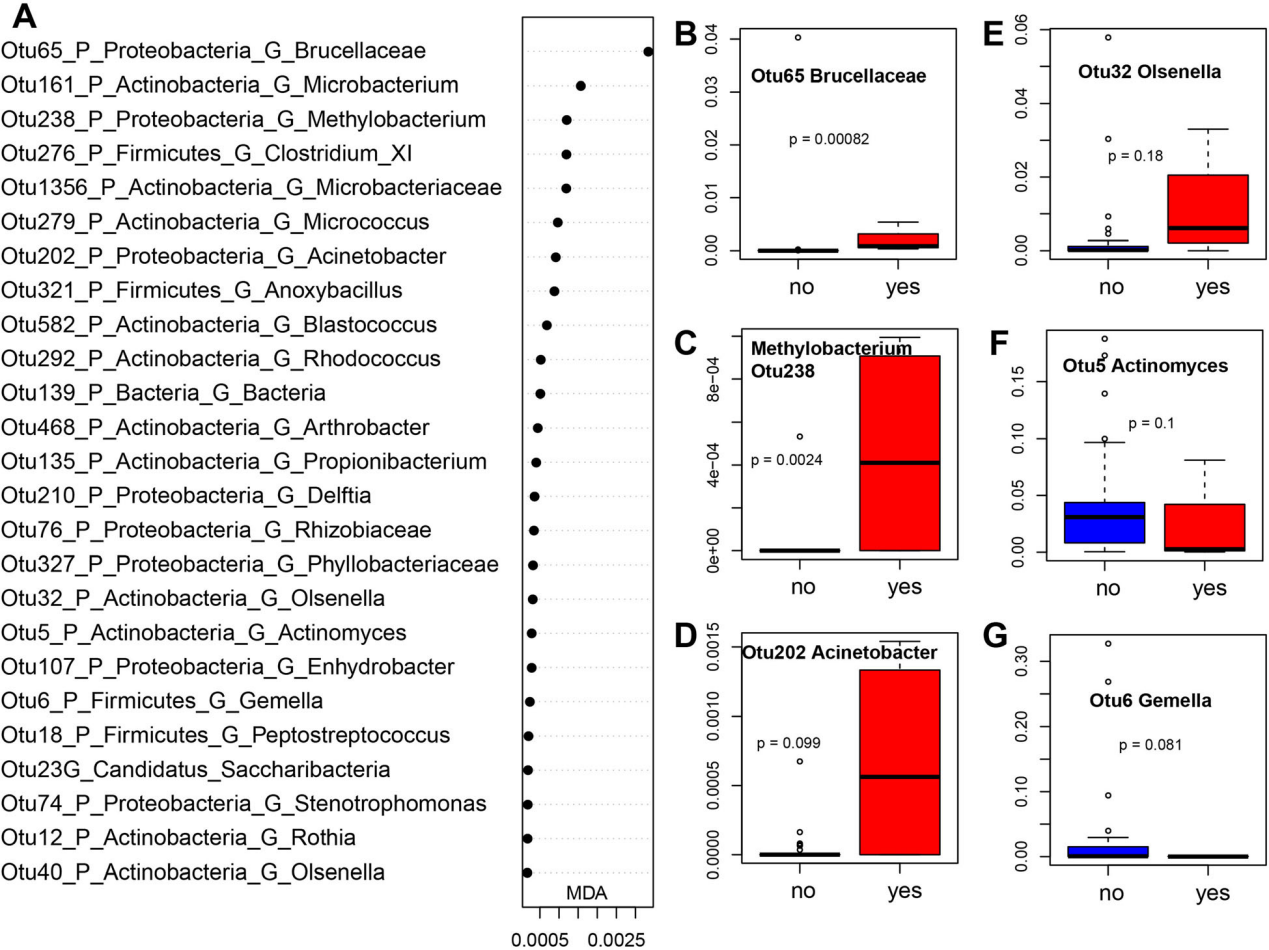
Bacterial OTUs that differentiate frequent (yes, 2–3 times) vs non-frequent (no, 0–1 time) AECOPDs: Mean Decrease in Accuracy plot of the top 15 bacterial OTUs (**a**); Box plots of representative OTUs (**b**-**g**). AUCRF was performed to find bacteria that differentiate frequent (yes, 2–3 times) vs non-frequent (no, 0–1 time) in AECOPD patients. A: top 25 bacterial OTUs were listed based on Mean Decrease in Accuracy (MDA) from AUCRF; **b**-**g**: Box plots of relative abundance of representative OTUs, and the p values were calculated by using Wilcoxon test. AECOPD: acute exacerbations of chronic obstructive pulmonary disease. AECOPD frequency (0&1) (29 samples) and AECOPD frequency (2&3) (4 samples) based on the times having AECOPD in the past 1 year

The frequency of previous AECOPD has also been implicated in the occurrence of AECOPD. The sputum microbiome of patients with 0 or 1 AECOPD had greater alpha diversity than those with 2 or 3 AECOPDs (Fig. 4b), although no significant separation in sputum microbiome membership or structure was detected between these subtypes (Figure S6 C and D). A random forest model identified bacterial OTUs that differentiate frequent (2 or more) vs. non-frequent (0 or 1) AECOPDs with an AUC of 0.966 (specificity 0.966, sensitivity 1.000) (Figure S4 C) using only two OTUs. Among these 20 top features, *Actinomyces* (OTU5) and *Gemella* (OTU6) were more abundant in non-frequent AECOPDs, while other OTUs were enriched in the frequent AECOPDs (Fig. 6 and Figure S8).

**Fig. 7:**
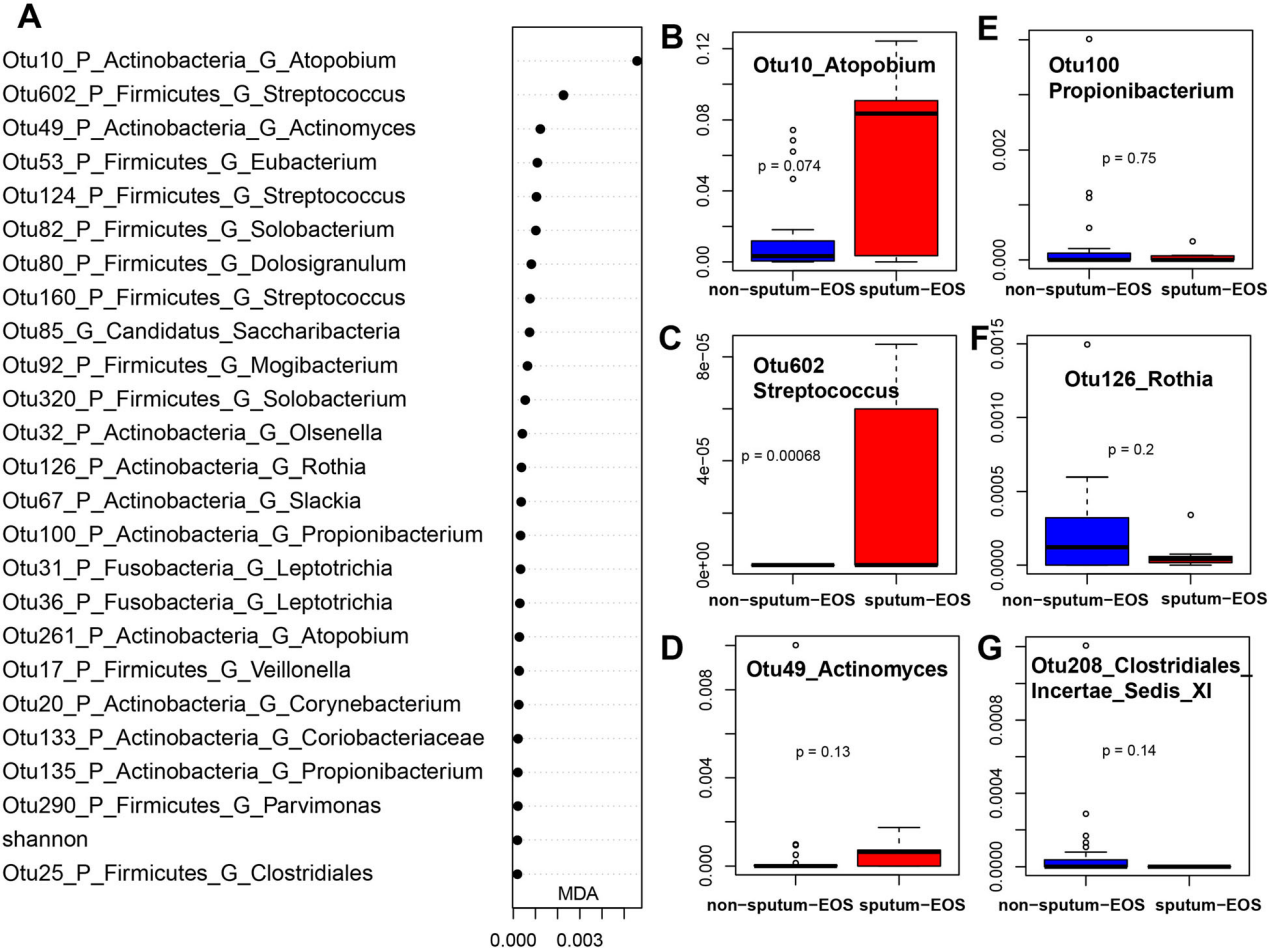
Bacterial OTUs that differentiate sputum-eosinophilic vs non-sputum-eosinophilic AECOPDs: Mean Decrease in Accuracy plot of the top 15 bacterial OTUs (**a**); Box plots of representative OTUs (**b**-**g**). AUCRF was performed to find bacteria that differentiate sputum eosinophilic and non-eosinophilic in AECOPD patients. . A: top 25 bacterial OTUs were listed based on Mean Decrease in Accuracy (MDA) from AUCRF; **b**-**g**: Box plots of relative abundance of representative OTUs, and the p values were calculated by using Wilcoxon test. AECOPD: acute exacerbations of chronic obstructive pulmonary disease. Sputum eosinophils < 3% as non-sputum-eosinophilic (26 samples), sputum eosinophils ≥3% sputum-eosinophilic (7 samples)

The sputum eosinophil concentration has also been used in AECOPD studies. Again, we did not observe any differences in alpha or beta diversity between sputum-eosinophilic or non-sputum-eosinophilic AECOPD subtypes (Fig. 4c, Figure S6E and F). However, we were able to differentiate the two subtypes based on a total of 23 bacterial OTUs with an AUC of 0.786 (specificity 0.857, sensitivity 0.769). Among the top 50 OTUs, *Atopobium* (OTU10, 261, 462), *Streptococcus* (OTU602, 160, 124), *Actinomyces* (OTU49), *Eubacterium* (OTU53), *Solobacterium* (OTU82, 85,320), *Mogibacterium* (OTU92), *Leptotrichia* (OTU31), *Slackia* (OTU67), *Clostridiales* (OTU25), and *Corynebacterium* (OTU20), were enriched in sputum-eosinophilic AECOPDs. The non-sputum-eosinophilic AECOPDs had more *Propionibacterium* (OTU100), *Prevotella* (OTU48, 304), *Selenomonas* (OTU119), *Olsenella* (OTU32), *Clostridiales_Incertae_Sedis_XI* (OTU208), *Treponema* (OTU142), *Coriobacteriaceae* (OTU179), *Parvimonas* (OTU290), *Microbacterium* (OTU161), *Rothia* (OTU126), and *Veillonella* (OTU17) (Fig. 7 and Figure S9).

## Discussion

With the development of next-generation sequencing, significant progress has been made in our understanding of the etiology of COPD [8, 12, 27–33]. However, most of these studies were focused on Caucasians; little is known about the sputum microbiome of COPD in the Chinese population. In this study, we characterized the sputum microbiome of sputa collected from the stable, AECOPD, and recovery stages of COPD patients and healthy controls from a Han population receiving care in the clinic of Peking University Third Hospital.

AECOPD has been the research focus of many scientists and clinicians because of its deleterious effects on lung function and the quality of life for COPD patients. In a recent study, Mayhew et al. investigated the longitudinal changes of the sputum microbiome between stable and AECOPD in the AERIS study. Due to the many confounders associated with sputum microbiome, such as disease stage, antibiotics, age and genetics, longitudinal studies are more powerful as the patients serve as their own controls. The authors did not find any difference in community alpha diversity or core bacterial taxa between the stable vs. the AECOPD states except the genus *Moraxella* [28]. In an independent study, Wang et al. characterized the sputum microbiome in the COPDMAP study in the United Kingdom. The stable microbiome was not different from the AECOPD microbiome in alpha diversity either [9]. Similarly, in other respiratory diseases such as cystic fibrosis, the stable microbiome also had comparable microbial diversity with exacerbation microbiomes [34]. Consistent with these studies, no significant difference in community diversity (Shannon index or richness) was observed between stable and AECOPD in the Chinese cohort of our study. These data suggest that it is likely the changes in the abundance of existing bacteria in the sputum rather than the gain or loss of certain bacterial species that cause the onset of AECOPD.

The dysbiosis of the sputum microbiome, shifting from a balanced composition to an imbalanced state dominated by one or a few bacterial species, has been implicated in AECOPD. In the COPDMAP study, Wang and colleagues measured the dysbiosis of AECOPD as a Z-score and found that 49 out of the 119 AECOPD with a Z-score greater than 2 showed significant dysbiosis [9]. In our study, while significant differences in sputum microbiome membership and structure between AECOPD and healthy controls were observed, only moderate differences in community structure between the AECOPD and stable microbiomes were detected (ANOSIM R = 0.29) with substantial overlaps. This inconsistency may be attributed to the heterogeneity of AECOPD, the small sample size of stable samples, and the cross-sectional nature of this study.

Despite many studies on the sputum microbiome in AECOPD in Caucasians, no consensus has been reached yet. In some studies, Proteobacteria has been reported to be the dominant phylum in the sputum microbiome of COPD [9] and members of Proteobacteria such as *Haemophilus* and *Moraxella* have been associated with the onset of AECOPD and disease severity [28]. Wang and colleagues observed a significant increase in the relative abundance of *Moraxella* in COPD versus healthy controls and during AECOPD. They also found *Moraxella* associated host responses primarily related to AECOPD [35]. In other studies, however, members of Firmicutes such as *Streptococcus* and *Veillonella* dominated the lung microbiome in COPD [8, 12, 28, 29]. Filho et al. found that *Streptococcu*s, *Prevotella* and *Veillonella* of the Firmicutes phylum were the top genera in the sputa of 102 patients hospitalized due to AECOPD. Compared to a one-year mortality data, survivors had a higher relative abundance of *Veillonella* while non-survivors were enriched with *Staphylococcus* [36]. In our study, *Streptococcus* was the most dominant bacteria in all categories (e.g., stable, AECOPD), followed by *Rothia*. We identified a list of bacterial OTUs that were able to differentiate AECOPD samples from other types. Most of these OTUs were less represented in AECOPD samples except *Pseudomonas* (OTU216, *P. aeruginosa*, Table S2), which was consistent with a recent study where Ren and colleagues showed that COPD patients with active *Streptococcus* or *Rothia* infections tended to have lower rates of AECOPD than patients with active *Pseudomonas* and patients with lower bacterial biomass [12]. These data suggest that although *Pseudomonas* is not as abundant in the COPD microbiome as in other lung diseases such as cystic fibrosis, it might play important roles in causing the onset of AECOPD. Given the huge population in China, it is difficult to evaluate how representative the sputum microbiome revealed in our study is. More studies of patients with AECOPD from different geographic locations of China are needed to examine the variation in sputum microbiome within the Chinese population and to determine the differences in sputum microbiome between eastern and western countries.

Due to its heterogeneity, it is important to classify the subtypes of AECOPD. Eosinophilic AECOPD has drawn increasing attention in recent years because blood eosinophil counts are correlated with AECOPD. However, until recently, little was known about the sputum microbiome of eosinophilic AECOPD. Wang et al. compared the sputum microbiome in different subtypes (e.g., bacterial, eosinophilic, viral, bacterial/eosinophilic) of AECOPD. They found pronounced differences between bacterial and eosinophilic exacerbations, with a significant decrease of alpha diversity and Firmicutes and an increase of Proteobacteria in the bacterial subtype. Notably, a significant decrease in *Streptococcus* and an increase in *Haemophilus* was observed in the bacterial subgroup [8]. In our study, we combined all the non-eosinophilic AECOPD as a group and developed a random forest model to identify bacterial taxa that differentiate eosinophilic AECOPD. Consistent with Wang et al., we detected a greater abundance of *Streptococcus* and *Fusobacterium* in the eosinophilic AECOPD; however, *Haemophilus* was also more abundant in the eosinophilic AECOPD. A different panel of bacterial OTUs was identified to differentiate sputum-eosinophilic AECOPD based on the sputum eosinophil count. A greater abundance of *Streptococcus* was also observed in the sputum-eosinophilic AECOPD.

This study has some limitations. First, the sample size is relatively small, especially for stable samples. Although the goals of most studies are to find biomarkers and/or triggers of AECOPD, it is critical to have stable samples to compare with. However, stable samples are more difficult to obtain because most patients visit clinics when they have increased symptoms, i.e., AECOPD. In addition, most of these samples were collected from patients from a single clinic in Beijing. A study with a large sample size collected from several centers is needed to better understand the sputum microbiome of COPD in China. Second, most of the samples were cross-sectional, collected from different patients. Due to the many factors confounding COPD sputum microbiome studies (e.g., age, gender, disease severity, gender, environment, and antibiotic usage), it is important to perform a longitudinal study comparing the stable and AECOPD samples collected from the same sets of patients. In that case, the patients serve as their own controls and rule out many of those confounders. Another limitation of this study is the lack of virus data, which, together with the microbiome data, explains some variations in the clinical status. Moreover, microbiome analysis only provides changes in relative abundance. Future studies are needed to quantify the total bacterial load by qPCR to determine the differences in absolute bacterial load of total and specific bacteria between groups of different clinical status in COPD. Of note, although sputum has been widely used to study the lung microbiome, considerable overlap between sputum microbiome and saliva microbiome has been observed [19]. As negative correlation between oral hygiene on COPD has been reported [20, 37], the spatial dynamics between upper and lower respiratory tract microbiome and the potential roles of these oral microbiome in COPD development in the Chinese population is highly desired.

## Conclusion

In this study, we characterized the sputum microbiomes of COPD patients collected during the stability, exacerbation, and recovery periods. *Streptococcus* was the most dominant genus among all different types of sputum in this Chinese cohort. We also identified microbiome biomarkers that differentiate subtypes of COPD exacerbations regarding eosinophilic counts and frequencies. These findings contribute to our understanding of the pathobiology of AECOPD in a Chinese cohort and provide insights into novel management of the different subtypes of AECOPD.

## Supplementary information

**Additional file 1: Figure S1.** Beta diversity of antibiotic-treated AECOPD microbiomes. **Figure S2.** Principal Coordinate Analysis of the lung microbiome structure based on the Bray-Curtis distance matrix. **Figure S3.** The sputum microbiome at the phylum level. Each bar shows the relative abundance of individual (A) or average (B) samples collected at AECOPD, healthy controls, recovery and stable. **Figure S4.** Random forest models developed by the AUC-RF package that differentiate AECOPD vs other samples (A), eosinophilic vs non-eosinophilic AECOPD (B), frequent vs non-frequent AECOPD (C) and sputum-eosinophilic vs non-sputum-eosinophilic AECOPD (D). The ‘Kopt’ shows the number of optimal variables fitted the AUCRF model. The values in parentheses are (specificity, sensitivity). **Figure S5.** Top 25 OTUs identified by AUCRF that differentiate AECOPD from other samples. **Figure S6.** PCoA plots showing the dissimilarity in community membership (Jaccard) and structure (Bray-Curtis) distance with respect to blood eosinophil count (A and B), frequency (C and D) and sputum eosinophil concentration (E and F). **Figure S7.** Boxplots of top25 bacterial OTUs predicting eosinophilic AECOPD. **Figure S8.** Boxplots of top 25 OTUs predicting the frequency of AECOPD. **Figure S9.** Boxplots of top 50 OTUs predicting sputum-eosinophilic AECOPD. **Table S1.** The composition of top 30 genera in each group. **Table S2.** The NCBI Blast of major OTUs related to Streptococcus and Pseudomonas.

## Data Availability

Raw data were deposited into the SRA database with accession number PRJNA647843.

## Abbreviations

COPD: Chronic obstructive pulmonary disease
AECOPD: Acute exacerbations of COPD;
ANOSIM: ANalysis Of SIMilarity
BMI: Body mass index
FEV: Forced expiratory volume
