# Supplementary material for: The sputum microbiome associated with different sub-types of AECOPD in a Chinese cohort
Source: BMC Infect Dis. 2020 Aug 18;20:610. doi: 10.1186/s12879-020-05313-y (PMC7433052; doi:10.1186/s12879-020-05313-y)
Supplement: Supplementary file 1 — Additional file 1: Figure S1. Beta diversity of antibiotic-treated AECOPD microbiomes. Figure S2. Principal Coordinate Analysis of the lung microbiome structure based on the Bray-Curtis distance matrix. Figure S3. The sputum microbiome at the phylum level. Each bar shows the relative abundance of individual (A) or average (B) samples collected at AECOPD, healthy controls, recovery and stable. Figure S4. Random forest models developed by the AUC-RF package that differentiate AECOPD vs other samples (A), eosinophilic vs non-eosinophilic AECOPD (B), frequent vs non-frequent AECOPD (C) and sputum-eosinophilic vs non-sputum-eosinophilic AECOPD (D). The ‘Kopt’ shows the number of optimal variables fitted the AUCRF model. The values in parentheses are (specificity, sensitivity). Figure S5. Top 25 OTUs identified by AUCRF that differentiate AECOPD from other samples. Figure S6. PCoA plots showing the dissimilarity in community membership (Jaccard) and structure (Bray-Curtis) distance with respect to blood eosinophil count (A and B), frequency (C and D) and sputum eosinophil concentration (E and F). Figure S7. Boxplots of top25 bacterial OTUs predicting eosinophilic AECOPD. Figure S8. Boxplots of top 25 OTUs predicting the frequency of AECOPD. Figure S9. Boxplots of top 50 OTUs predicting sputum-eosinophilic AECOPD. Table S1. The composition of top 30 genera in each group. Table S2. The NCBI Blast of major OTUs related to Streptococcus and Pseudomonas. [file 12879_2020_5313_MOESM1_ESM.docx]

**SUPPLEMENTARY INFORMATION**

**The lung microbiome associated with different sub-types of AECOPD in a Chinese cohort**

**Juan Wang****^1^, Jianmin Chai^2^, Lina Sun^1^, Chun Chang^1^* and Jiangchao Zhao^2^***

1 Department of Respiratory and Critical Care Medicine, Peking University Third Hospital, Beijing, China

2 Department of Animal Science, Division of Agriculture, University of Arkansas, Fayetteville, AR, 72701, USA

**Figure S1**. Beta diversity of antibiotic-treated AECOPD microbiomes

**Figure S2**. Principal Coordinate Analysis of the lung microbiome structure based on the Bray-Curtis distance matrix.

**Figure S3**. The sputum microbiome at the phylum level. Each bar shows the relative abundance of individual (A) or average (B) samples collected at AECOPD, healthy controls, recovery and stable.

**Figure S4**. Random forest models developed by the AUC-RF package that differentiate AECOPD vs other samples (A), eosinophilic vs non-eosinophilic AECOPD (B), frequent vs non-frequent AECOPD (C) and sputum-eosinophilic vs non-sputum-eosinophilic AECOPD (D). The ‘Kopt’ shows the number of optimal variables fitted the AUCRF model. The values in parentheses are (specificity, sensitivity).

**Figure S5**. Top 25 OTUs identified by AUCRF that differentiate AECOPD from other samples.

**Figure S6**. PCoA plots showing the dissimilarity in community membership (Jaccard) and structure (Bray-Curtis) distance with respect to blood eosinophil count (A and B), frequency (C and D) and sputum eosinophil concentration (E and F)

**Figure S7**. Boxplots of top25 bacterial OTUs predicting eosinophilic AECOPD.

**Figure S8**. Boxplots of top 25 OTUs predicting the frequency of AECOPD

**Figure S9**. Boxplots of top 50 OTUs predicting sputum-eosinophilic AECOPD

**Table S1**. The composition of top 30 genera in each group

**Table S2**. The NCBI Blast of major OTUs related to Streptococcus and Pseudomonas

Figure S1 Beta diversity of antibiotic-treated AECOPD microbiomes


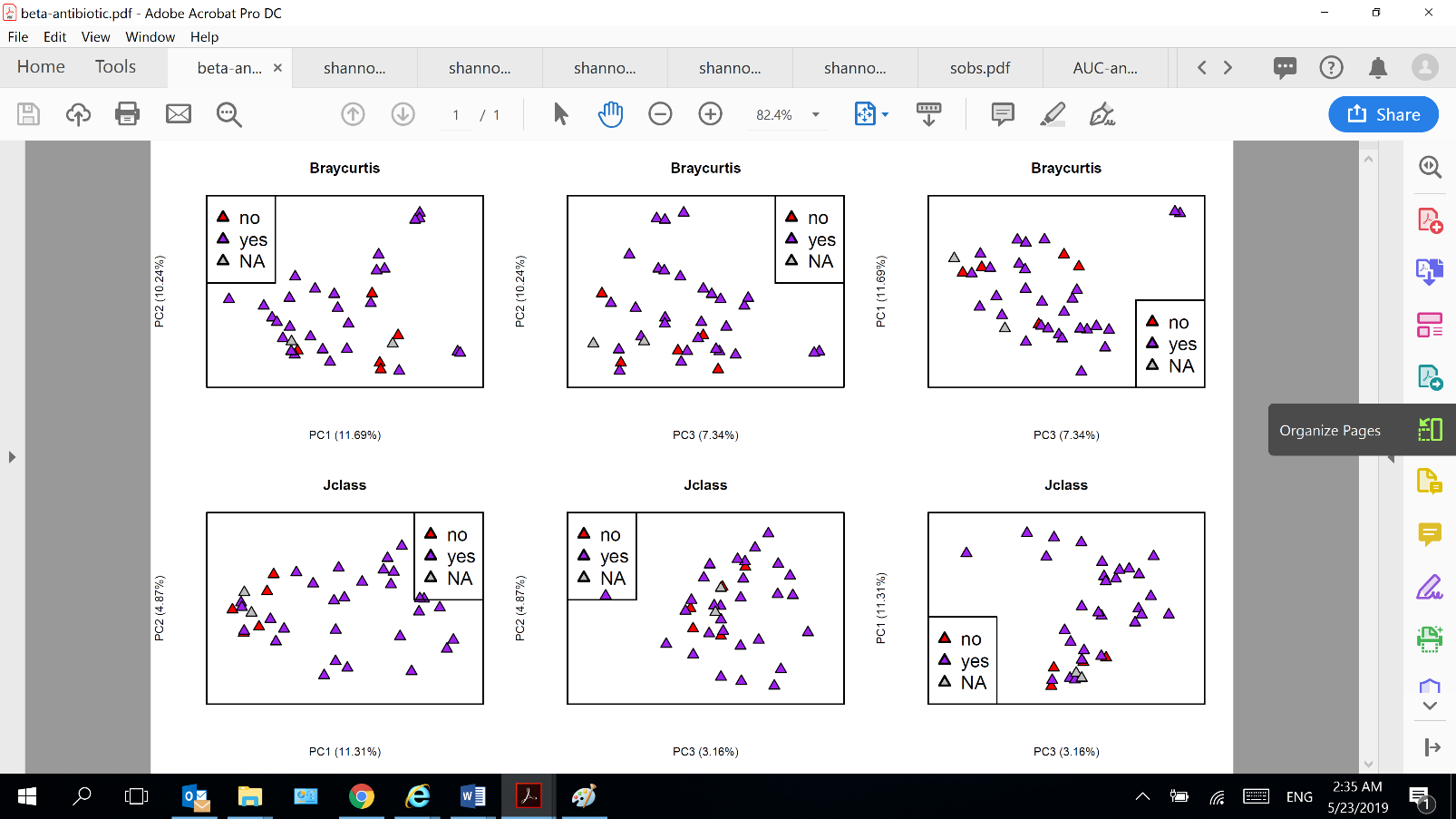


If AECOPD patient received antibiotics, ‘yes’ was labeled in figure. The ‘NA’ represented the missing antibiotic-treated information. AECOPD: acute exacerbations of chronic obstructive pulmonary disease.

Figure S2 Beta diversity AECOPD, Bray-Curtis distance


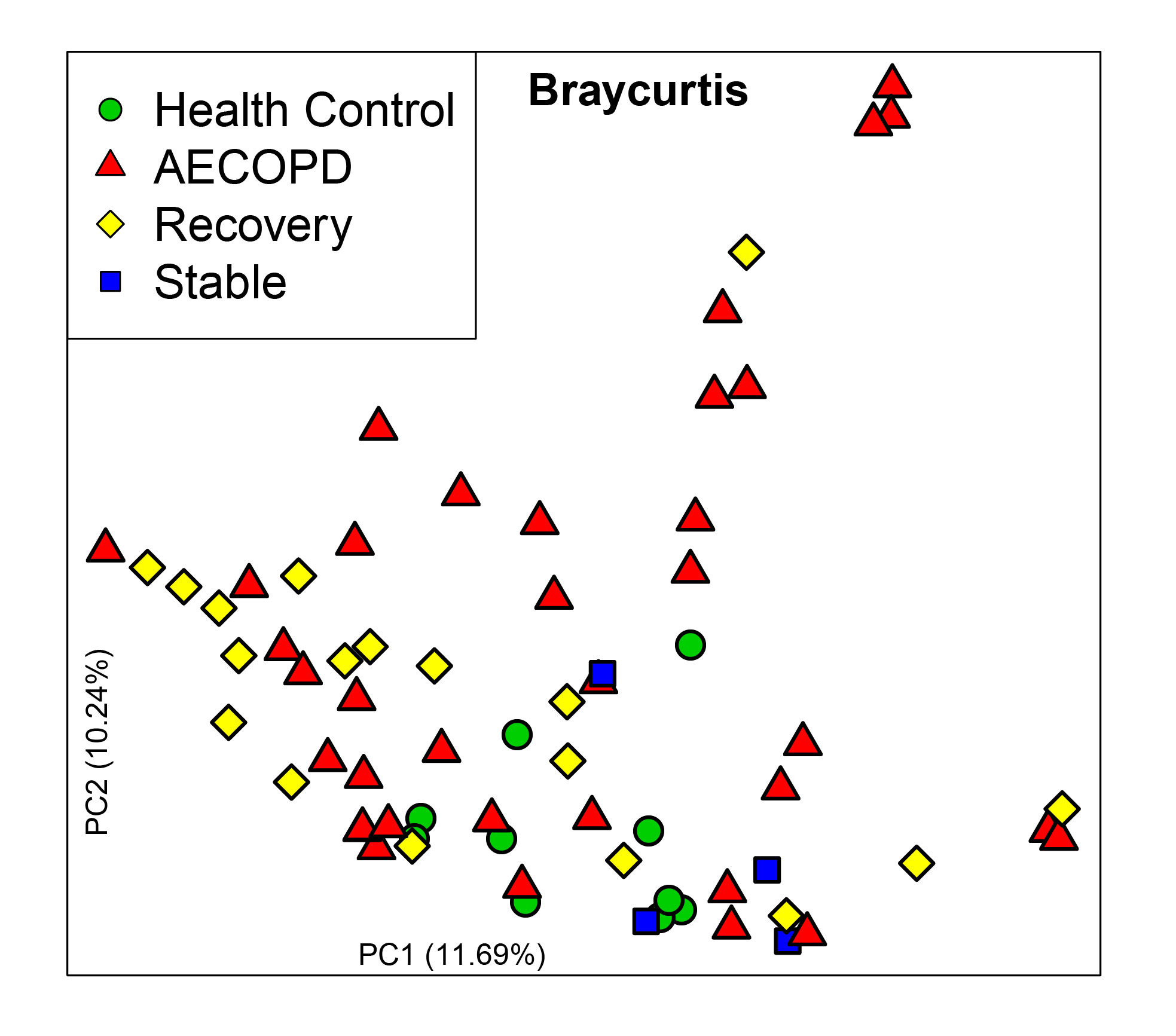


Figure S2. Principal Coordinate Analysis of the sputum microbiome structure based on the Bray-Curtis distance matrix.

Each point represents 1 subject with Health Control as green circle, AECOPD as red triangle and Stable as blue square. With respect to community structure, significant differences between AECOPD vs stable (R=0.22, P<0.05), healthy control vs recovery (R=0.44, P<0.05), and stable vs recovery (R=0.43, P<0.05) were revealed by the PCoA plot based on Bray-Curtis distance.

Health Control: subjects with no any clinical signs; AECOPD: acute exacerbations of chronic obstructive pulmonary disease; Recovery: the patient recovering from exacerbation treatment; Stable: stable period over 8 weeks free of an AECOPD.

Figure S3, Phylum level bar chart


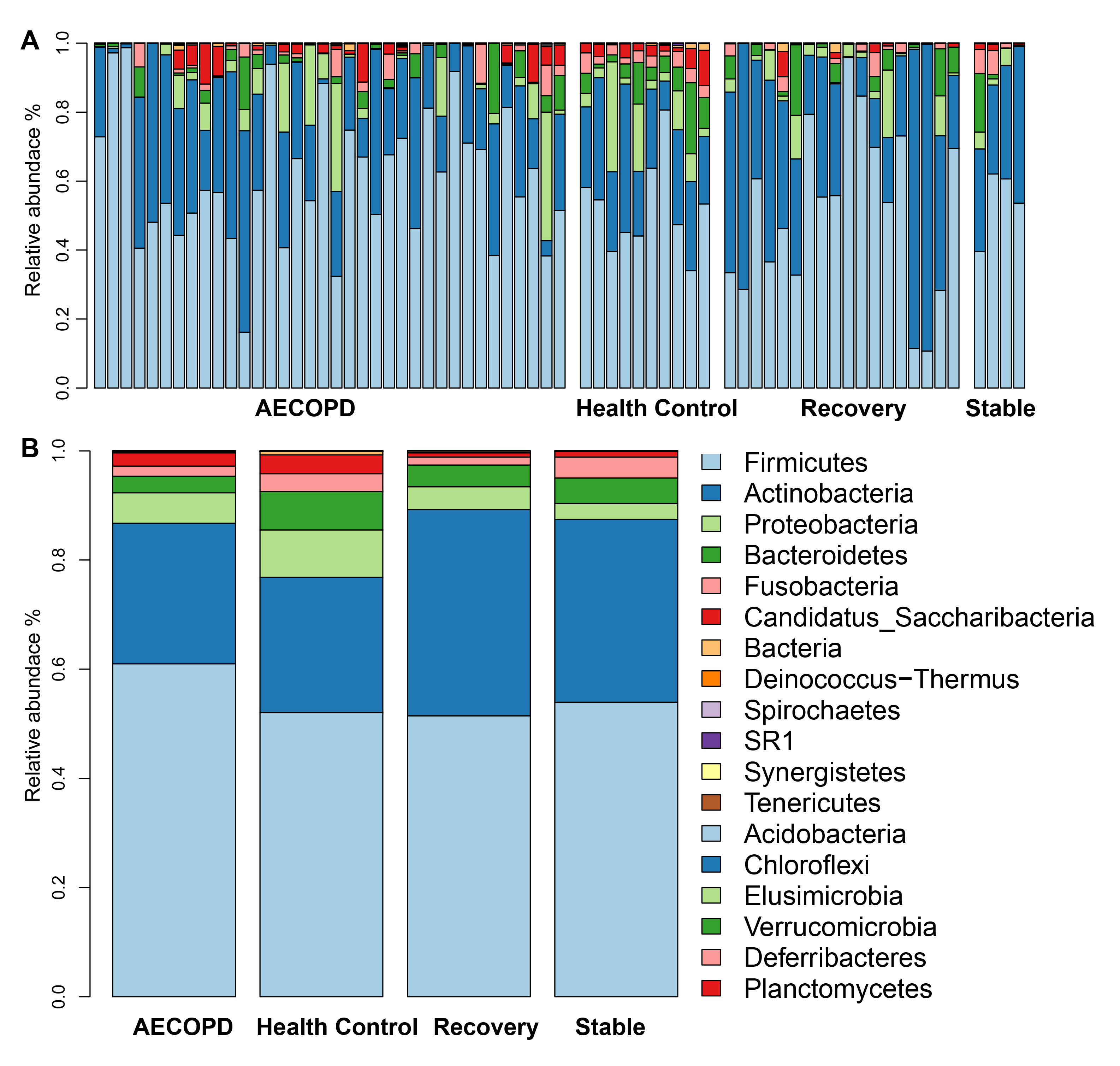


Figure S3. The sputum microbiome at the phylum level. Each bar shows the relative abundance of individual (A) or average (B) samples collected at AECOPD, healthy controls, recovery and stable.

Health Control: subjects with no any clinical signs; AECOPD: acute exacerbations of chronic obstructive pulmonary disease; Recovery: the patient recovering from exacerbation treatment; Stable: stable period over 8 weeks free of an AECOPD

Figure S4. AUC curve


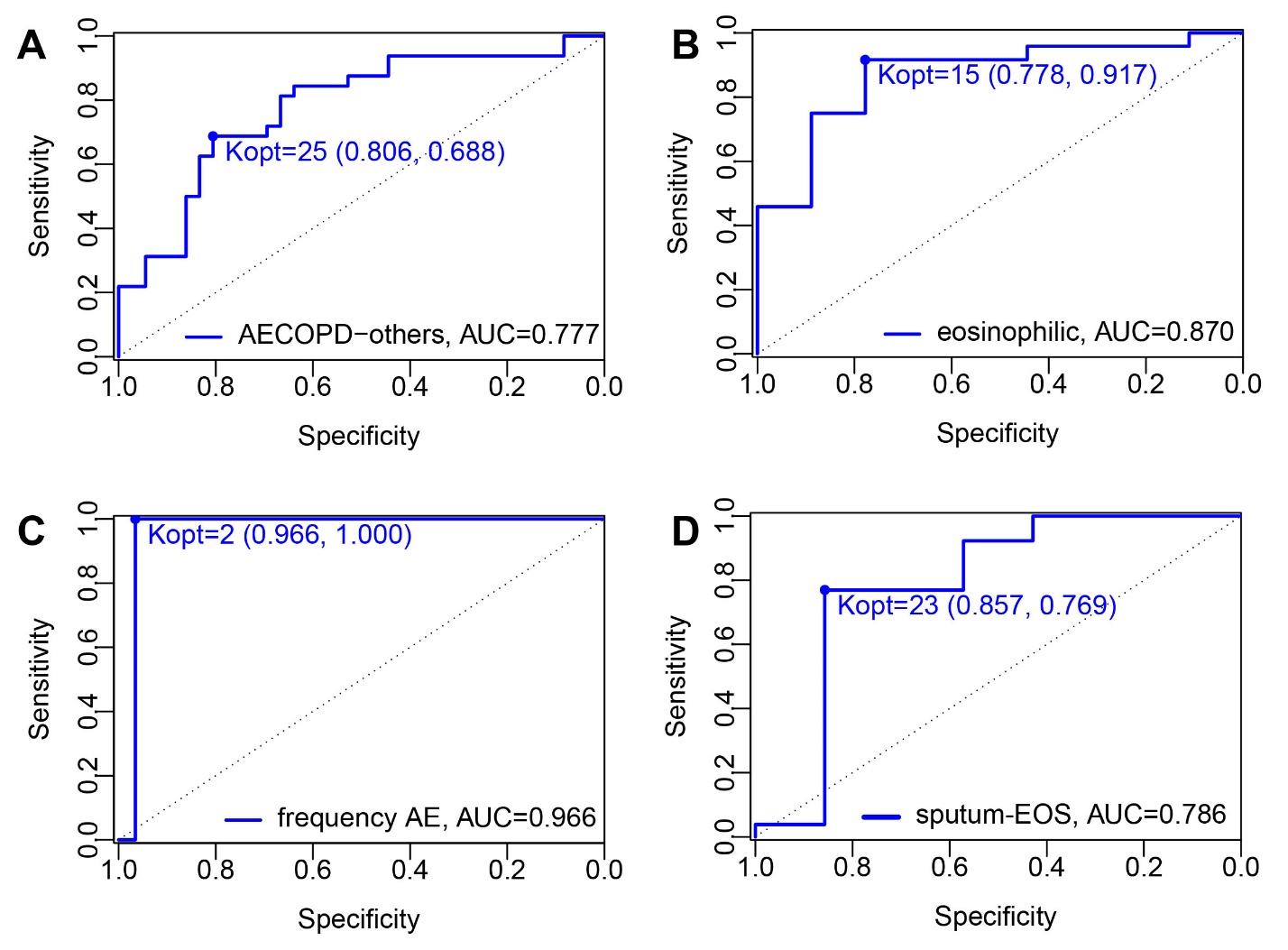


Figure S4. Random forest models developed by the AUC-RF package that differentiate AECOPD vs other samples (A), eosinophilic vs non-eosinophilic AECOPD (B), frequent vs non-frequent AECOPD (C) and sputum-eosinophilic vs non-sputum-eosinophilic AECOPD (D). The ‘Kopt’ shows the number of optimal variables fitted the AUCRF model. The values in parentheses are (specificity, sensitivity).

Others were a combination of health control, recovery and stable samples. Health Control: subjects with no any clinical signs; AECOPD: acute exacerbations of chronic obstructive pulmonary disease; Recovery: the patient recovering from exacerbation treatment; Stable: stable period over 8 weeks free of an AECOPD

Figure S5.


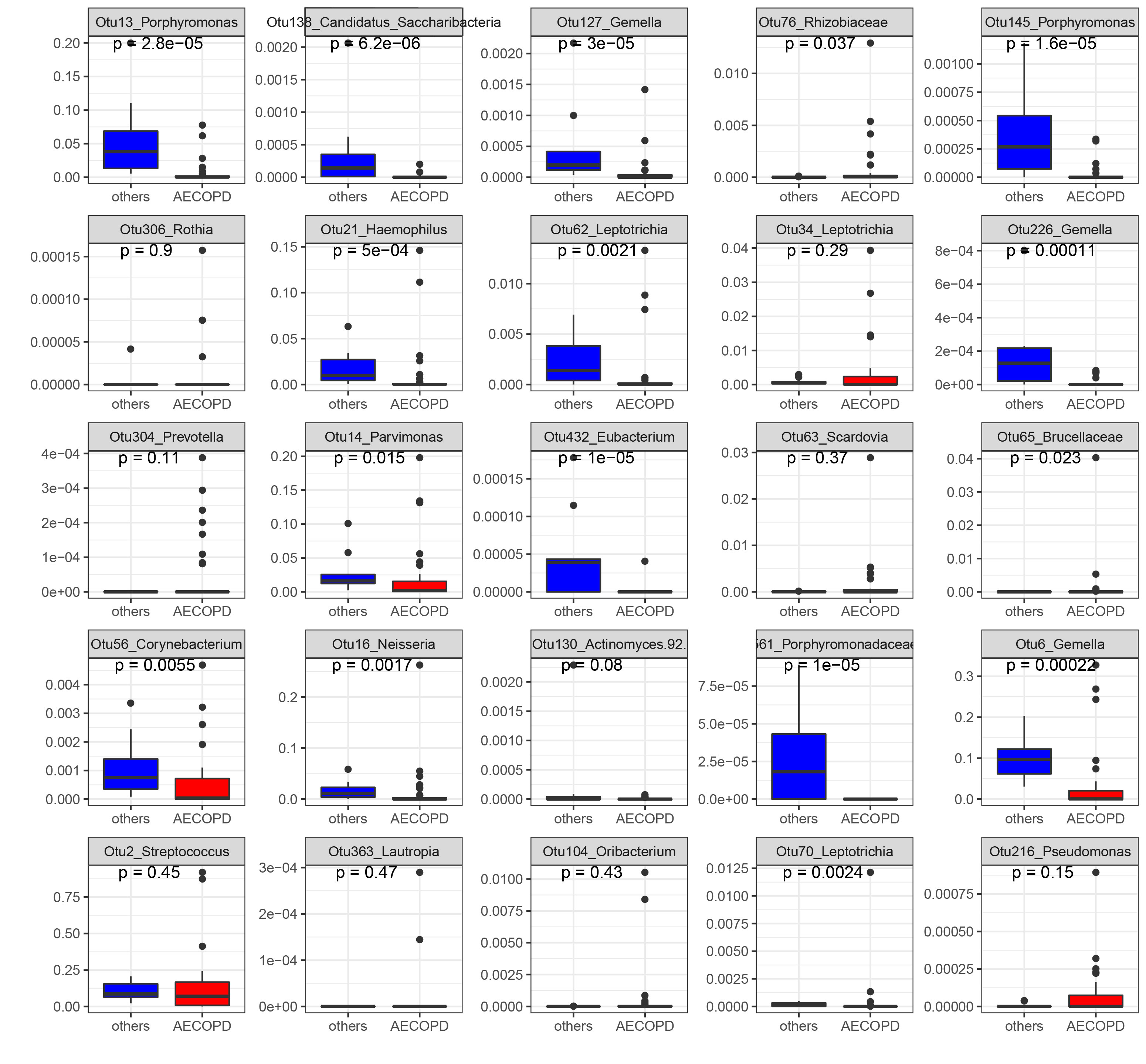


Figure S5. Top 25 OTUs identified by AUCRF that differentiate AECOPD from other samples.

Others were a combination of health control, recovery and stable samples. The p values were calculated by using Wilcoxon test.

Health Control: subjects with no any clinical signs; AECOPD: acute exacerbations of chronic obstructive pulmonary disease; Recovery: the patient recovering from exacerbation treatment; Stable: stable period over 8 weeks free of an AECOPD

Figure S6. PCoA plots showing the dissimilarity in community membership (Jaccard) and structure (Bray-Curtis) distance with respect to blood eosinophil count (A and B), frequency (C and D) and sputum eosinophil concentration (E and F)


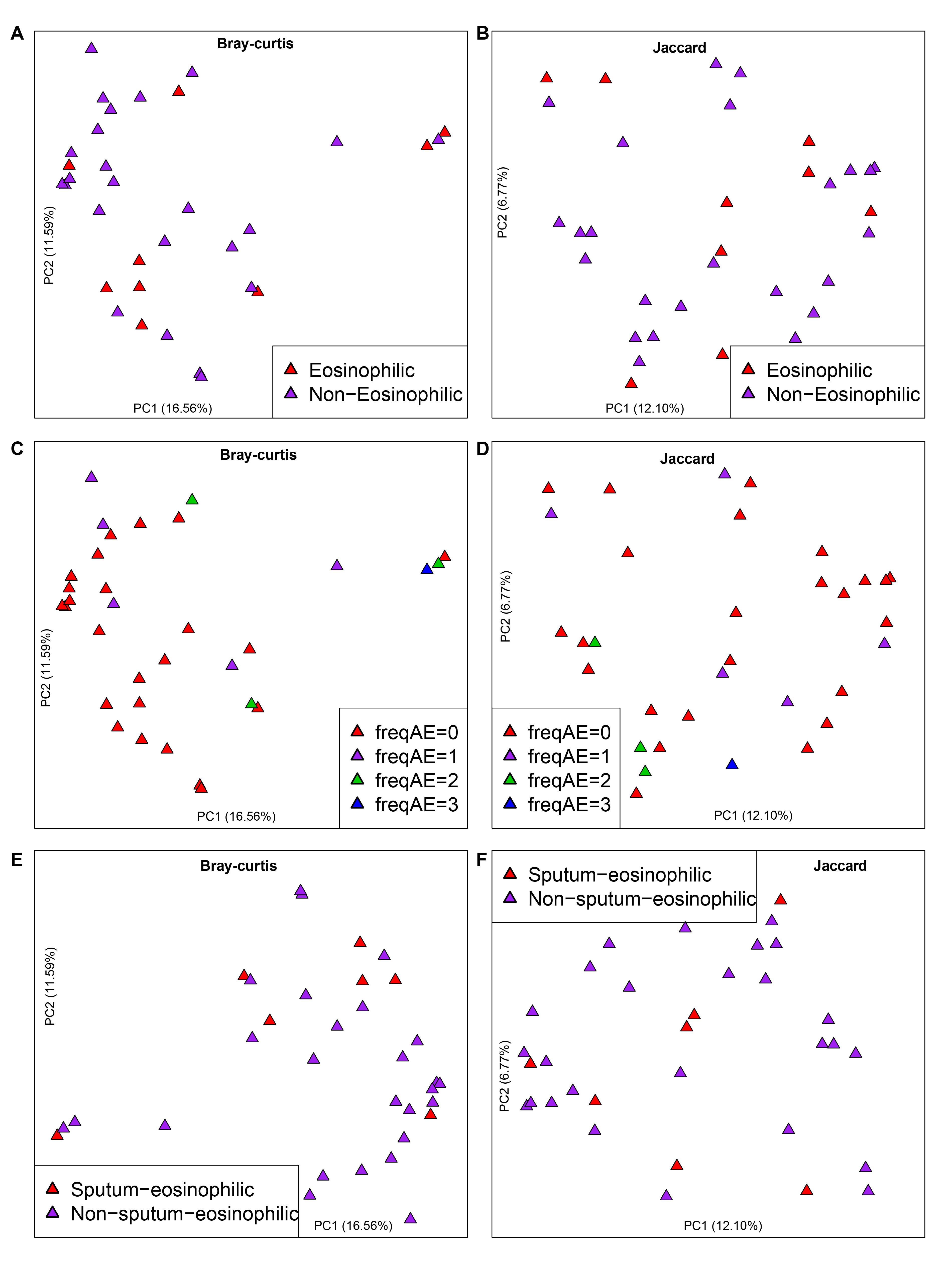


Figure S7. Boxplots of top25 bacterial OTUs predicting eosinophilic AECOPD.


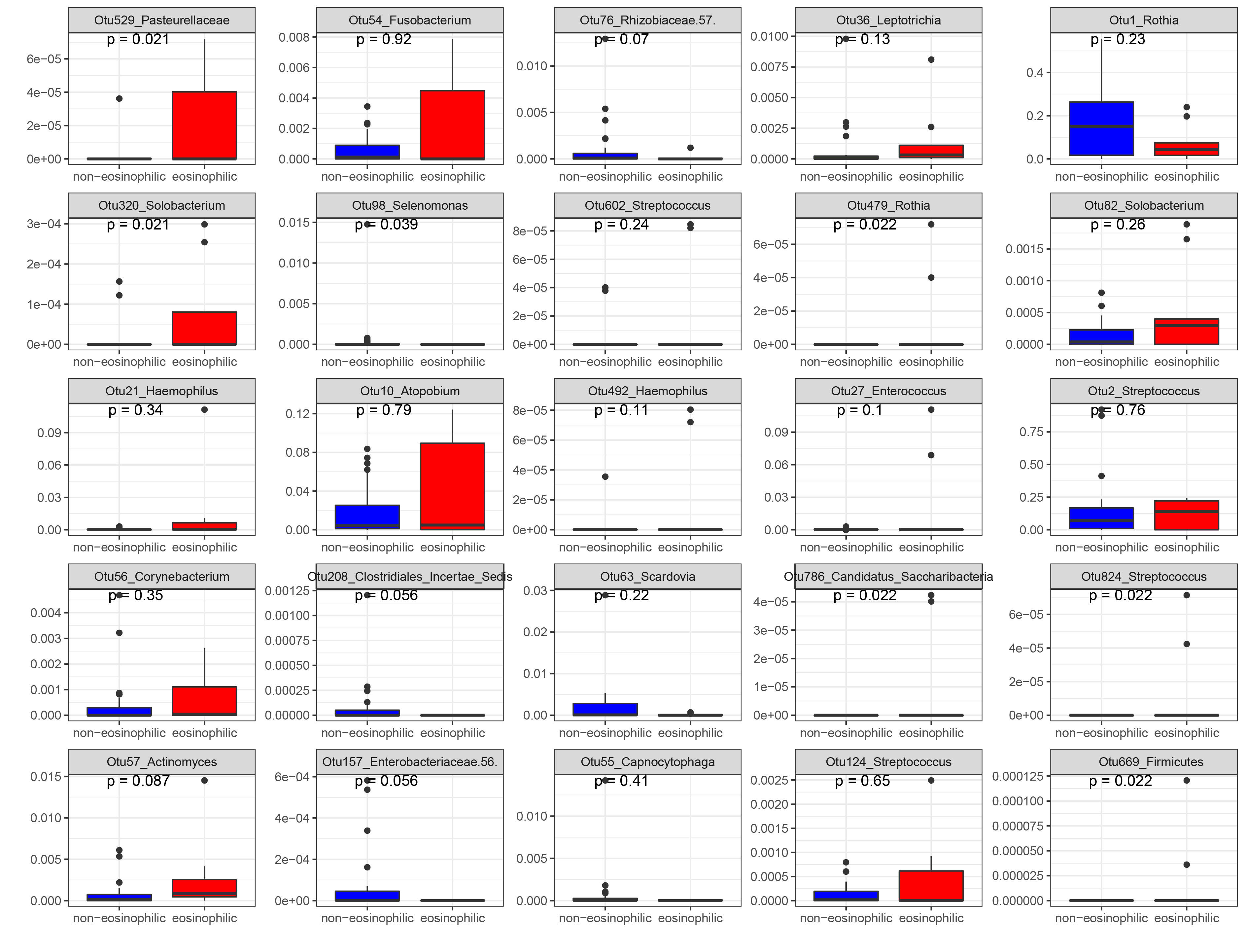


AUCRF was performed to find bacteria that differentiate serum eosinophilic and non-eosinophilic in AECOPD patients. The p values were calculated by using Wilcoxon test

Figure S8. Boxplots of top 25 OTUs predicting the frequency of AECOPD


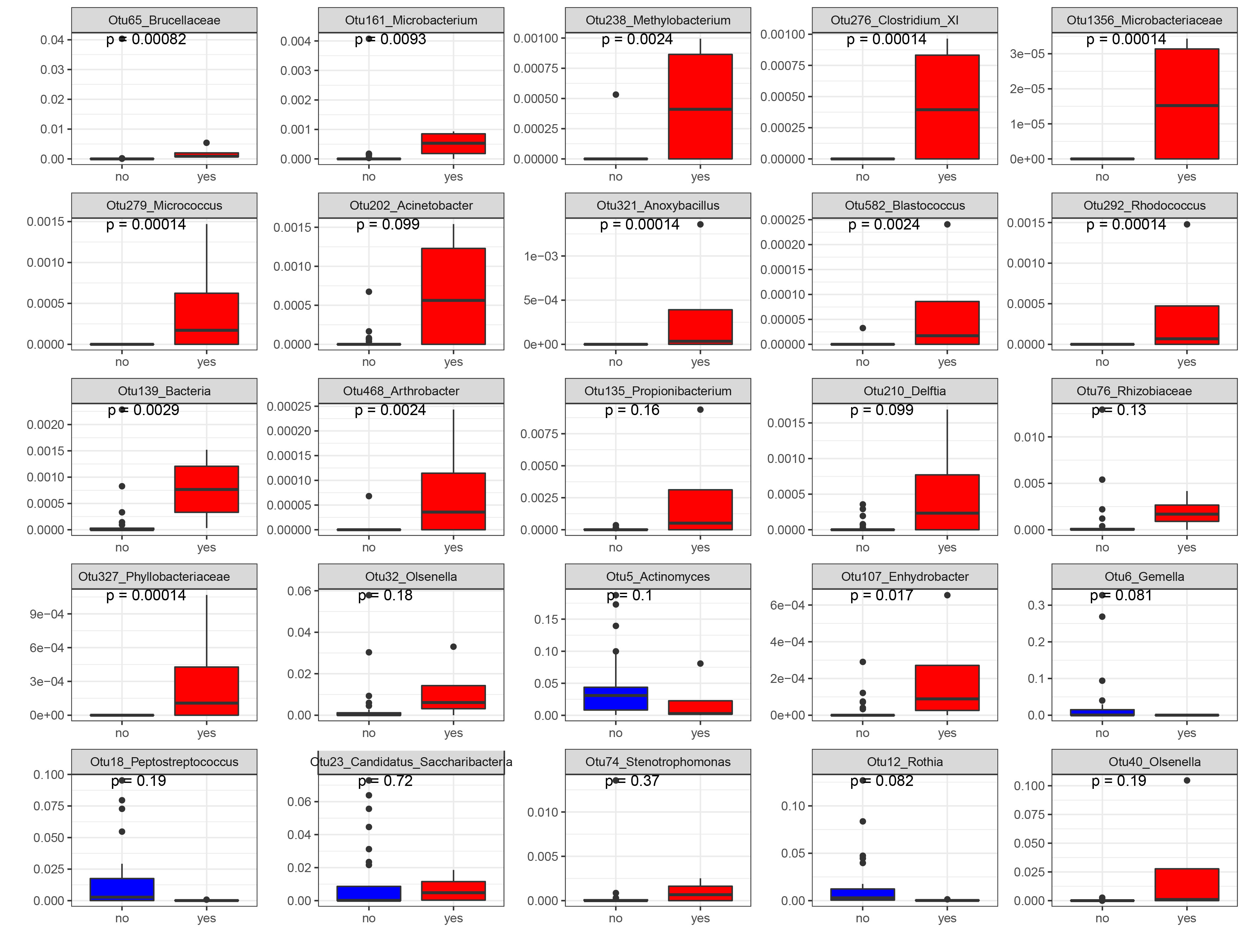


AUCRF was performed to find bacteria that frequent (2-3 times) vs non-frequent (0-1 time) AECOPDs in AECOPD patients. The p values were calculated by using Wilcoxon test

Figure S9. Boxplots of top 50 OTUs predicting sputum-eosinophilic AECOPD


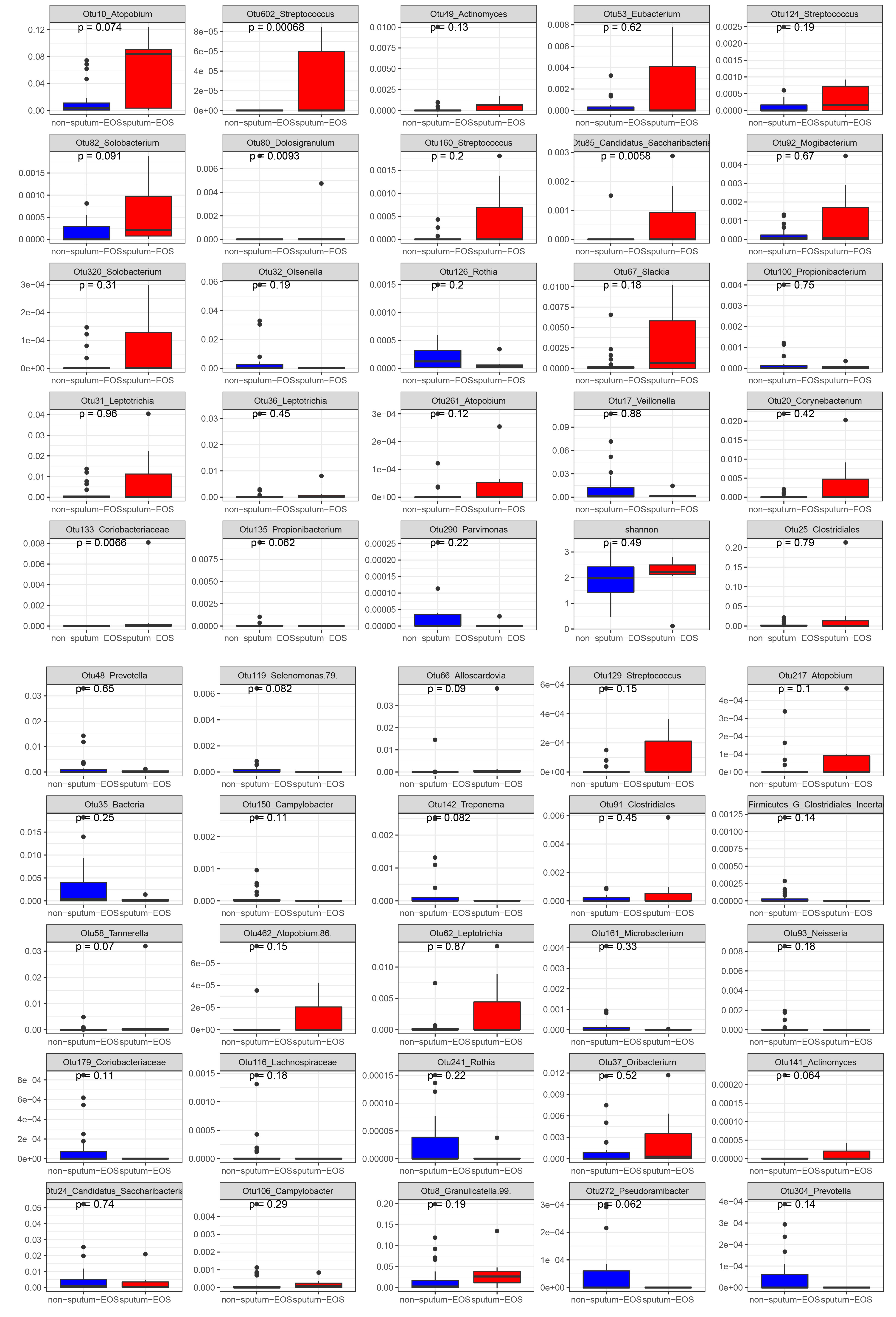


AUCRF was performed to find top 50 bacteria that differentiate sputum eosinophilic and non-eosinophilic in AECOPD patients. The p values were calculated by using Wilcoxon test.

Table S1 the composition of top 30 genera in each groups

| genera | Health Control | AECOPD | Recovery | Stable | pvalue-KW test |
| --- | --- | --- | --- | --- | --- |
| Streptococcus | 0.2752 | 0.2659 | 0.3660 | 0.3449 | 0.2897 |
| Rothia | 0.1293 | 0.1607 | 0.2620 | 0.2104 | 0.1945 |
| Actinomyces | 0.0660 | 0.0423 | 0.0771 | 0.0343 | 0.1306 |
| Staphylococcus | 0.0000 | 0.0783 | 0.0186 | 0.0152 | 0.0391 |
| Gemella | 0.1013 | 0.0339 | 0.0220 | 0.1243 | 0.0001 |
| Abiotrophia | 0.0012 | 0.0589 | 0.0420 | 0.0002 | 0.3936 |
| Granulicatella | 0.0412 | 0.0283 | 0.0246 | 0.0138 | 0.0799 |
| Neisseria | 0.0649 | 0.0233 | 0.0167 | 0.0158 | 0.0048 |
| Lactobacillus | 0.0000 | 0.0434 | 0.0040 | 0.0013 | 0.0100 |
| Atopobium | 0.0432 | 0.0231 | 0.0164 | 0.0175 | 0.0762 |
| Candidatus_Saccharibacteria | 0.0345 | 0.0244 | 0.0078 | 0.0101 | 0.0069 |
| Porphyromonas | 0.0592 | 0.0064 | 0.0207 | 0.0391 | 0.0010 |
| Leptotrichia | 0.0303 | 0.0172 | 0.0137 | 0.0361 | 0.0182 |
| Parvimonas | 0.0279 | 0.0207 | 0.0039 | 0.0080 | 0.0004 |
| Veillonella | 0.0207 | 0.0192 | 0.0058 | 0.0004 | 0.0072 |
| Peptostreptococcus | 0.0204 | 0.0131 | 0.0073 | 0.0143 | 0.0549 |
| Corynebacterium | 0.0045 | 0.0067 | 0.0172 | 0.0626 | 0.1431 |
| Lautropia | 0.0012 | 0.0115 | 0.0139 | 0.0042 | 0.4363 |
| Haemophilus | 0.0189 | 0.0097 | 0.0049 | 0.0076 | 0.0023 |
| Prevotella | 0.0052 | 0.0122 | 0.0075 | 0.0020 | 0.8453 |
| Clostridiales | 0.0054 | 0.0106 | 0.0048 | 0.0046 | 0.0124 |
| Capnocytophaga | 0.0033 | 0.0081 | 0.0062 | 0.0031 | 0.3303 |
| Olsenella | 0.0027 | 0.0100 | 0.0020 | 0.0016 | 0.0325 |
| Enterococcus | 0.0000 | 0.0051 | 0.0082 | 0.0000 | 0.1112 |
| Weissella | 0.0000 | 0.0063 | 0.0000 | 0.0000 | 0.5810 |
| Bacteria | 0.0061 | 0.0026 | 0.0032 | 0.0011 | 0.0372 |
| Oribacterium | 0.0035 | 0.0042 | 0.0011 | 0.0014 | 0.0076 |
| Lachnospiraceae | 0.0017 | 0.0043 | 0.0007 | 0.0007 | 0.0989 |
| Selenomonas | 0.0006 | 0.0042 | 0.0002 | 0.0004 | 0.0619 |
| Coriobacteriaceae | 0.0003 | 0.0038 | 0.0005 | 0.0015 | 0.3986 |
| Campylobacter | 0.0003 | 0.0032 | 0.0016 | 0.0004 | 0.9811 |

Health Control: subjects with no any clinical signs; AECOPD: acute exacerbations of chronic obstructive pulmonary disease; Recovery: the patient recovering from exacerbation treatment; Stable: stable period over 8 weeks free of an AECOPD

Table S2 The NCBI Blast of major OTUs related to *Streptococcus* and *Pseudomonas*

| OTUs | NCBI blast |
| --- | --- |
| Otu2_P_Firmicutes_G_Streptococcus | Streptococcus mitis |
| Otu3_P_Firmicutes_G_Streptococcus | Streptococcus sinensis |
| Otu11_P_Firmicutes_G_Streptococcus | Streptococcus salivarius |
| Otu22_P_Firmicutes_G_Streptococcus | Streptococcus dysgalactiae |
| Otu30_P_Firmicutes_G_Streptococcus | Streptococcus anginosus |
| Otu61_P_Firmicutes_G_Streptococcus | Streptococcus mutans |
| Otu114_P_Firmicutes_G_Streptococcus | Streptococcus rubneri |
| Otu120_P_Firmicutes_G_Streptococcus | Streptococcus parasanguinis |
| Otu124_P_Firmicutes_G_Streptococcus | Streptococcus australis |
| Otu160_P_Firmicutes_G_Streptococcus | Streptococcus sobrinus |
| Otu602_P_Firmicutes_G_Streptococcus | Streptococcus parasanguinis |
| Otu21_P_Proteobacteria_G_Haemophilus | Haemophilus parainfluenzae |
| Otu216_P_Proteobacteria_G_Pseudomonas | Pseudomonas aeruginosa |
